# Are Anesthesiology Providers Good Guessers? Heart Rate and Oxygen Saturation Estimation in a Simulation Setting

**DOI:** 10.1155/2019/5914305

**Published:** 2019-07-21

**Authors:** Efrain Riveros Perez, Enoe Jimenez, Camila Albo, Yashi Sanghvi, Nianlan Yang, Alexander Rocuts

**Affiliations:** ^1^Assistant Professor, Department of Anesthesiology and Perioperative Medicine, Medical College of Georgia at Augusta University, 1120 15th Street, Augusta, GA, USA; ^2^Outcomes Research Consortium, Cleveland Clinic, Cleveland, OH, USA; ^3^Research Assistant, Department of Anesthesiology and Perioperative Medicine, Medical College of Georgia at Augusta University, Augusta, GA, USA; ^4^Medical Student, Medical College of Georgia at Augusta University, Augusta, GA, USA; ^5^Undergraduate Student, Biomedical Engineering, Cornell University, Ithaca, NY, USA; ^6^Post-doctoral fellow, Department of Anesthesiology and Perioperative Medicine, Medical College of Georgia at Augusta University, Augusta, GA, USA

## Abstract

**Background:**

Anesthesia providers may need to interpret the output of vital sign monitors based on auditory cues, in the context of multitasking in the operating room. This study aims to evaluate the ability of different anesthesia providers to estimate heart rate and oxygen saturation in a simulation setting.

**Methods:**

Sixty anesthesia providers (residents, nurse anesthetics, and anesthesiologists) were studied. Four scenarios were arranged in a simulation context. Two baseline scenarios with and without waveform visual aid, and two scenarios with variation of heart rate and/or oxygen saturation were used to assess the accuracy of the estimation made by the participants.

**Results:**

When the accurate threshold for the heart rate was set at less than 5 beats per minute, the providers only had a correct estimation at two baseline settings with visual aids (*p*=0.22 and 0.2237). Anesthesia providers tend to underestimate the heart rate when it increases. Providers failed to accurately estimate oxygen saturation with or without visual aid (*p*=0.0276 and 0.0105, respectively). Change in recording settings significantly affected the accuracy of heart rate estimation (*p* < 0.0001), and different experience levels affected the estimation accuracy (*p*=0.041).

**Conclusion:**

The ability of anesthesia providers with different levels of experience to assess baseline and variations of heart rate and oxygen saturation is unsatisfactory, especially when oxygen desaturation and bradycardia coexist, and when the subject has less years of experience.

## 1. Background

Anesthesiology providers care for patients while performing multiple tasks. In such situations, reliance on vital sign monitoring is of utmost importance to avoid errors [1]. A vital sign monitor assists the anesthesia provider to execute complex decision-making processes and, at the same time, alerts the clinician of potentially life-threatening events that may occur while performing procedures. Attentiveness in anesthesia is essential as lack of monitor vigilance is commonly cited as a factor implicated in critical incidents in anesthesia practice [2]. Pulse oximetry monitors yield crucial information for patient care by means of a combination of oxygen saturation and heart rate integrated to plethysmography. Currently, this monitor is considered standard of care in anesthetic practice [3]. Data obtained by the pulse oximeter are displayed visually and in audible form. The result is a continuous mapping of numerical values related to comprehensive auditory dimensions. Sonification of information obtained from the pulse oximeter, a method to represent data by sounds, is delivered in the form of auditory cues that signal changes in oxygen saturation [4]. Therefore, changes in oxygen saturation values are perceived as variable pitch tones. Simultaneously, the rate of the output tones provides information regarding heart rate and rhythm.

Anesthesiologists perceive changes in physiologic parameters based on the auditory information conveyed by the pulse oximeter. The dynamic sound of the falling saturation enables providers to rapidly respond to a clinical problem. However, clinicians have difficulty identifying specific oxygen saturation values from the auditory tone alone [5]. Practitioners are able to detect the direction and pitch differences secondary to oxygen saturation drops, but not the magnitude of the change or the absolute saturation percentage number. In addition, acoustic properties of current pulse oximeters are not standardized across manufacturers and models [6]. New generation monitors are equipped with enhanced auditory displays that improve sound perception and facilitate clinical judgment of the output [7]. Heart rate information is continuously displayed on pulse oximetry monitors with reasonable accuracy [8]. Research studies addressing interpretation of sonification of heart rate tones and pitch cues by clinicians are scarce.

We propose this study to test the hypothesis that the ability of anesthesia providers with different experiences to detect changes in oxygen saturation and heart rate changes is unsatisfactory and that this discriminatory ability is worse with less years of experience.

## 2. Methods

After approval by the Institutional Board Review at Augusta University, sixty anesthesiology providers (residents, nurse anesthetists and anesthesiologists) were invited to participate in this study. This research complied with the American Psychological Association Code of Ethics. Those with auditory limitations or disorders were excluded. Participants signified their voluntary intent to participate by signing a university-approved informed consent document. Each participant was evaluated in a quiet room, where different audio clips of heart tones and oxygen saturation were played (supplementary material (available here)). The audio clips contained a series of recordings obtained from a human patient simulator (CAE Healthcare, Montreal, Quebec, Canada) connected to a Philips MMS IntelliVue X2 (Philips Medical System, BG Eindhoven, the Netherlands) and a Nellcor™ Bedside Respiratory Patient Monitoring System PM1000N (Medtronic, Minneapolis, MN, USA).

Four scenarios were created. Scenario A was used as a baseline, consisting of a heart rate of 70 beats per minute and oxygen saturation of 100%, without visual aid (waveforms and numerical values). In scenario B, progressive heart rate increases of five beats per minute every three seconds from baseline with stable oxygen saturation of 100% occurred. Scenario C was a baseline scenario with the same parameters of scenario A, with the addition of visual aid (waveforms only). Scenario D used baseline values of 60 beats per minute for heart rate and 97% for oxygen saturation, followed by simultaneous drops in heart rate and oxygen saturation. Rates of decrease of these variables were five beats per minute every three seconds and 2% every three seconds, respectively. The series of recordings were arranged in the following sequential order: A, B, C, B, A, D, C, and D. We evaluated accuracy of the participants to detect baseline values and changes in variables during conduction of the four scenarios.

### 2.1. Statistical Analysis

All statistical analyses were performed using SAS 9.4 (SAS Institute Inc. Cary, NC, USA). Statistical significance was assessed at an alpha level of 0.05 unless otherwise noted. Descriptive statistics of the 60 participants were determined within each level of training experiences. One-way ANOVA was used to compare the distribution of age among the different levels, and chi-squared tests were used to compare the distributions of gender and music experience. One-sample *t*-tests were performed on the absolute values of the difference between the estimation and true heart rate or oxygen saturation values (Estimation—True value) at different recording settings to determine whether the estimation was accurate or not. For heart rate estimation, an absolute value within 5 beats per minute was considered accurate. For oxygen saturation, an offset value less than 2% was considered accurate. Repeated measures mixed models were used to examine differences between the different settings of records of heart rate and oxygen saturation for accuracy estimation. Each model included fixed effects of recording settings, visual aid, and training levels, as well as the two-and three-factor interactions between record settings, visual aid, and training levels. An unstructured correlation structure was used. Post hoc pairwise comparisons between different recording settings with or without visual aid were determined using a Bonferroni adjustment to the overall alpha level to control the number of comparisons made.

## 3. Results

Sixty anesthesiology providers with an average age of 33.4 ± 8.54 were recruited. Demographic characteristics are displayed in Table 1. Difference in age among different levels of training experiences was detected (*p*=0.0001), with the group of attending anesthesiologists being the oldest. Gender distribution was also different among different levels of experience (*p*=0.0002). Among the 28 female providers, nurse anesthetists (CRNA) represented the largest proportion (60.71%), while among the 32 male providers, third-year anesthesia residents (CA3) represented the largest proportion (50.00%). There was no significant difference on prior music experience among the different groups.

**Figure 1:**
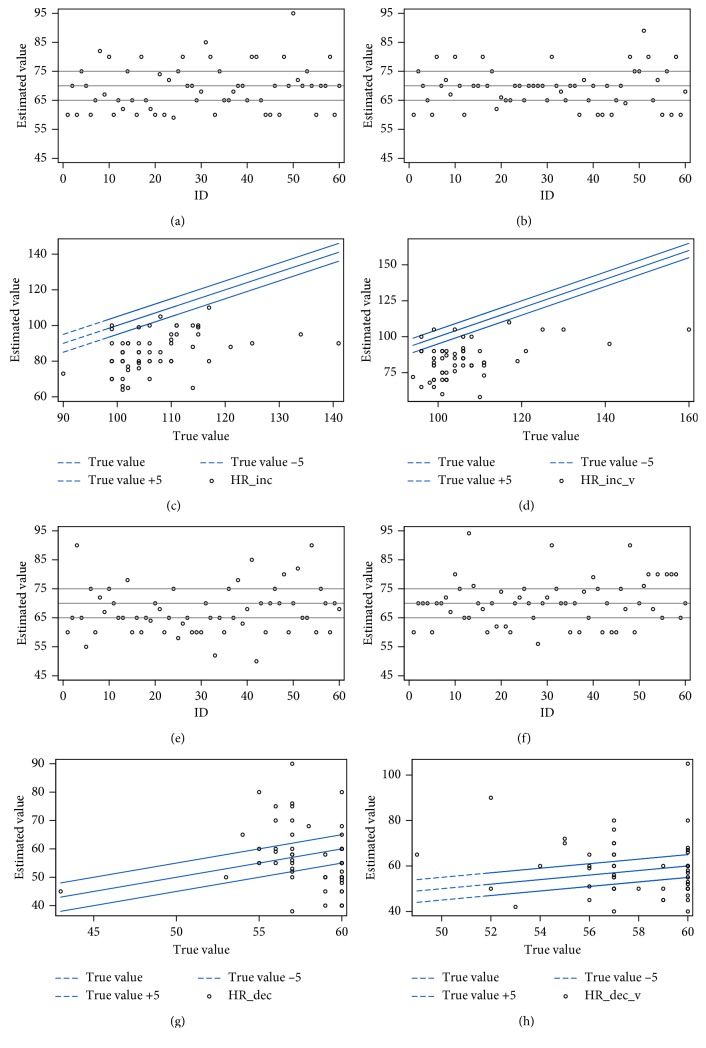
Heart rate estimation under different recording settings. Each dot represents one individual estimation, and the lines represent the true values and the acceptable correct estimating values (true value ±5). (a) Scenario A first attempt. (b) Scenario C first attempt. (c) Scenario B after A. (d) Scenario B after C. (e) Scenario A second attempt. (f) Scenario C second attempt. (g) Scenario D after A. (h) Scenario D after C.

**Figure 2:**
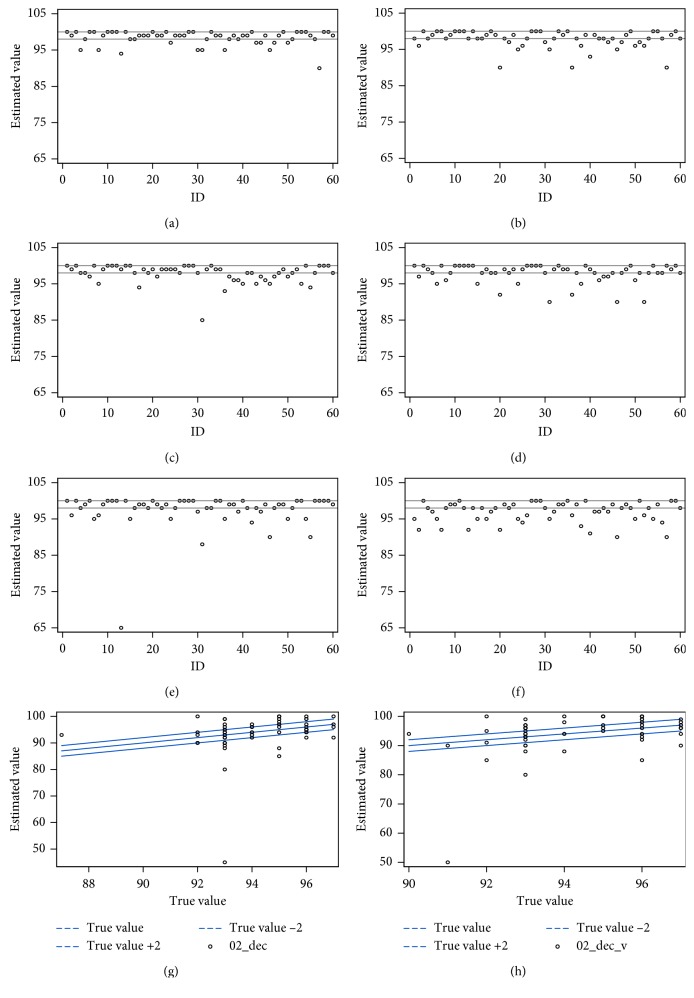
Oxygen saturation estimation under different recording settings. Each dot represents one individual estimation, and the lines represent the true values and the acceptable correct estimating values (true value ±2). (a) Scenario A first attempt. (b) Scenario C first attempt. (c) Scenario B after A. (d) Scenario B after C. (e) Scenario A second attempt. (f) Scenario C second attempt. (g) Scenario D after A. (h) Scenario D after C.

To avoid the compensation of overestimation to underestimation, we determined estimation accuracy using the absolute values of the difference between the true value and the estimated value (Table 2). In general, the providers' estimations of heart rate were not satisfactory. When the accurate threshold for the heart rate was set at less than 5 beats per minute, the providers only had a correct estimation at two baseline settings with visual aids (*p*=0.22 and 0.2237). The threshold to assess accuracy of oxygen saturation estimation was arbitrarily set at 2% because the study was done with a change of a percentage decrease drop of 2% for every new change in the heart rate. As shown in Figure 1, it appears that anesthesia providers tend to underestimate or overestimate the heart rate when it increases. For oxygen saturation estimation, data show that providers did a fine job when saturation was kept stable at 100% along with either a stable or increased heart rate. However, when oxygen saturation was decreasing in the presence of drops in the heart rate, the providers failed to accurately estimate it with or without visual aid (*p*=*0*.0276 and 0.0105, respectively) (Table 3 and Figure 2).

**Figure 3:**
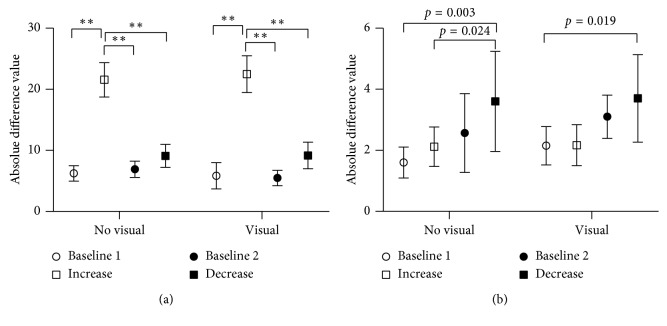
Pairwise comparison of (a) heart rate and (b) SaO_2_ estimation accuracy under different recording settings (^*∗∗*^ *p* < 0001).

When potential covariates such as different recording settings, experience levels, and prior music experience were considered, only the change in recording settings significantly affected the accuracy of heart rate estimation (ndf = 3, ddf = 413, *F* = 158.12, *p* < 0.0001). The estimation accuracy was worst when providers listened to increasing heart rates (Table 2 and Figure 3). The results for oxygen saturation estimation accuracy (Table 4) were similarly affected by different settings (ndf = 3, ddf = 413, *F* = 8.79, *p* < 0.0001). On the other hand, different experience levels affected the estimation accuracy (ndf = 3, ddf = 55, *F* = 2.94, *p*=0.041), with interns exhibiting the worst performance on estimation of oxygen saturation.

## 4. Discussion

Our study shows that the accuracy of anesthesia providers with different levels of experience to detect changes in heart rate and oxygen saturation using auditory cues in a simulated setting is not satisfactory, with the caveat that our results apply to devices/sound tracks and traced parameters employed in our design. Furthermore, although baseline normal values of oxygen saturation can be discriminated by auditory assessment, the ability of anesthesia providers to accurately determine the level of oxygen saturation when this variable is dropping is deficient, especially when it is accompanied by bradycardia.

Anesthesia providers perform multiple tasks on patients during critical times in the operating room. Some procedures performed by the anesthesiologist require a combination of mental concentration and visual focus. The split attention of the anesthesiologist during these procedures may lead to a decreased level of vigilance over vital variables [9]. Anesthesia providers may be able to detect changes in direction but not in magnitude when pulse oximeter values change [10]. Selective attention entails a process of prioritization of information to discriminate between attended and unattended data [11], and theoretical and empirical evidence support the notion that suppression of unattended information is necessary to focus attention [12]. Anesthesiologists depend on auditory signal processing when they are unable to watch the vital sign monitor. Our results evidence that relying on auditory cues to identify deviation from normal values of oxygen saturation and heart rate is all but deficient. Lack of attention to changes in vital signs can be the result of fatigue [13], work overload [14], stress [15], inexperience, or insufficient training in the identification of differential pitch sounds [16]. We conducted the study in a simulated setting and on rested individuals to eliminate the effects of stress and fatigue. We also evaluated providers with different levels of experience. In our opinion, training to identify the different tones of heart rate and oxygen saturation is necessary, as pitch sounds vary among different manufacturers.

Hypoxemia is one of the most dreaded outcomes an anesthesiologist can encounter during the intraoperative period. The widespread use of pulse oximetry in the developed world has paralleled a significant decrease in anesthesia-related mortality [17]. Despite the use of pulse oximetry, intraoperative hypoxemia continues to occur and is associated with immediate and perioperative negative consequences [18]. Detection of changes in oxygen saturation based on auditory cues by the human ear depends on the physiology of the auditory system, the attention of the receiver of the signal, and the way the sound output is presented by the monitor. As the frequency of a sound changes, so does its subjective pitch. Pitch is the subjective property of a sound that helps the receiver to categorize its quality along a linear perceptual dimension. Pitch is described in terms of frequency and can be measured using a musical scale. The human ear can detect a signal sound between 20 and 20.000 Hz. Over a range of frequencies between 500 and 4000, a healthy human being can detect 0.5% of variation in tonal frequency [19]. Loeb et al. studied 21 pulse oximeters evidencing fundamental frequencies at 100% oxygen saturation ranging between 479 and 921 Hz, and between 38 and 404 Hz at 1% oxygen saturation [20]. Since at low oxygen saturation, a percentage variation in pitch is small for the frequency range, the pitch of pulse tones might not be perceived as decreasing at low saturation levels. However, at high saturation levels, the pitch difference may be significant. Our study shows that decreases in oxygen saturation are not accurately diagnosed by participants, despite a favorable range of frequencies at oxygen saturations above 90%. We argue that the limitation to accuracy does not lie primarily on physiological barriers, but rather on insufficient training in identifying the pitch differences. On the other hand, different manufacturers use different algorithms to define pitch steps as saturation declines, with some of them using pitch change per percentage unit and some others per interval. We think that in order to enhance the pitch difference and facilitate detection of desaturation by auditory cues, the use of large intervals (i.e., 5% saturation change) would be beneficial. The participants in our study were overall inaccurate to detect changes in both heart rate and oxygen saturation; however, their accuracy further deteriorated as values deviated from baseline. This finding is relevant as baseline detection has less clinical application compared to detection of dynamic changes in a critical situation.

We did not find any relationship between musical experiences and accuracy of detection of cardiac and pulse oximetry tones. Liang et al. demonstrated that musicians outperform nonmusicians in detecting frequency changes in quiet and noisy conditions, measured by electroencephalographic response [21]. Auditory training utilizes neural plasticity to increase sensitivity to acoustic information under changing frequencies, by means of overlapping neural networks [22]. We consider these findings might support a recommendation to train anesthesia providers to recognize frequency changes associated with differential pitch conveyed by pulse oximeters. Finally, familiarity of the receiver with the pitch sound may play a relevant role in the ability to discriminate sounds with different tonal frequencies. Krishnan et al. showed that activity within the rostral brainstem, called frequency following response (FFR), was elicited by four Mandarin tones in native Chinese speakers in contrast with their native English-speaking counterparts [23]. These findings may suggest that “training of the brain” may be used to identify specific pitch sounds like those associated with changes in oxygen saturation. Further research is necessary to elucidate the mechanisms underlying this phenomenon.

Our study has limitations. Simulation settings differ from real-life operating room situations in the variety of auditory stimuli overlapping at different points in time. Extrapolation of our findings to the operating room is limited; however, we argue that the lack of accuracy of anesthesia providers to estimate heart rate and oxygen saturation variations may be exaggerated in the operating room, where distractions are present. We studied anesthesia providers, which may limit the application of our results to other areas where vital signs monitors are employed. In addition, the heart rate and oxygen saturation tones vary widely among device manufacturers, in the absence of a universally accepted standard. As a result, our findings must be taken cautiously when apparatus/sound tracks generated by other devices are traced. Furthermore, our study did not assess the effect of ambient noise or changes in volume of the monitor. These factors are potentially important in a real-life situation. We did not evaluate occupational dimensions of the anesthesia provider such as fatigue or stress. Future research is necessary to evaluate the association between the accuracy of clinicians to assess heart tones and pulse oximeter output by auditory cues and incidence of near-misses and adverse outcomes. Conduction of experimental studies in real operating room situations is necessary to analyze the impact of human (fatigue, stress, concentration, and auditory acuity) and environmental factors (noise, distractions, and tone volume) on rapid detection of physiological changes displayed by electronic devices.

In conclusion, the ability of anesthesia providers with different levels of experience to assess baseline and variations of heart rate and oxygen saturation is unsatisfactory. Estimation of the variation in these parameters is even worse, especially when oxygen desaturation and bradycardia coexist, and when the subject has less years of experience. These findings are only generalizable in the context of controlled devices/sound tracks and traced parameters employed in our study.

## Figures and Tables

**Table 1:** Descriptive statistics.

	Overall	Attending (*N* = 10)	Resident (*N* = 23)	CRNA (*N* = 20)	Intern (*N* = 7)	*p* value
*N* (%)	60 (100)	10 (16.67)	23 (38.33)	20 (33.33)	7 (11.67)	
Age, mean (SD)	33.4 (8.54)	45.8 (10.04)	33.3 (3.82)	37.1 (8.87)	31.0 (6.51)	0.0001^*∗*^
Gender, *N* (%)						**0.0002** ^*∗∗*^
Female	28 (46.67)	2 (7.14)	7 (25.00)	17 (60.71)	2 (7.14)	
Male	32 (53.33)	8 (25.00)	16 (50.00)	3 (9.38)	5 (15.63)	
Music experience, *N* (%)						**0.5894** ^*∗∗*^
Yes	17 (28.33)	2 (11.76)	9 (52.94)	5 (29.41)	1 (5.88)	
No	43 (71.67)	8 (18.60)	14 (32.56)	15 (34.88)	6 (13.95)	

^*∗*^One-way ANOVA. ^*∗∗*^Chi-squared test. SD, standard deviation; CRNA, nurse anesthetist.

**Table 2:** Estimation accuracy of heart rate and oxygen saturation under different recording settings.

Settings	Absolute value of heart rate difference			Absolute value of oxygen saturation		
	Mean	SD	*p* value^*∗*^	Mean	SD	*p* value^*∗∗*^
Scenario A first attempt	6.2	4.88	0.0274	1.6	1.97	0.9397
Scenario B after A	21.5	10.84	<0.0001	2.1	2.49	0.3590
Scenario C first attempt	5.8	8.35	0.22	2.2	2.42	0.3165
Scenario B after C	22.5	11.66	<0.0001	2.2	2.59	0.3101
Scenario A second attempt	6.9	5.15	0.003	2.6	5.00	0.1918
Scenario D after A	9.1	7.30	<0.0001	3.6	6.33	0.0276
Scenario C second attempt	5.5	4.89	0.2237	3.1	2.75	0.0015
Scenario D after C	9.2	8.42	0.0002	3.7	5.56	0.0105

^*∗*^One-sample *t*-tests with the null hypothesis that *u* ≤ 5. ^*∗∗*^One-sample *t*-tests with the null hypothesis that *u* ≤ 2. SD; standard deviation.

**Table 3:** Repeated measures mixed model results for recording setting, music, and training level on heart rate estimation accuracy.

Effect	Scenario	Visual aid	Experience level	Music experience	Adjusted LS mean	SE	*F*	*p* value
Setting	Scenario A				−1.1	1.20	158.12	<0.0001
	Scenario B				−21.6	1.20		
	Scenario C				−1.2	1.20		
	Scenario D				−0.5	1.2		

Visual aid		Scenario C			−5.7	1.05	0.79	0.3760
		Scenario A			−6.5	1.05		

Experience level			Attending		−5.7	1.94	1.13	0.3432
			Resident		−6.1	1.25		
			CRNA		−8.4	1.39		
			Intern		−4.2	2.32		

Music experience				Yes	−6.7	1.56	0.50	0.4820
				No	−5.5	0.96		

Visual aid × setting^*∗*^	Scenario C	Yes			−1.6	1.45	1.26	0.2870
	Scenario B				−21.9	1.45		
	Scenario C				0.06	1.45		
	Scenario D				0.54	1.45		
	Scenario A	No			−0.52	1.45		
	Scenario B				−21.3	1.45		
	Scenario A				−2.5	1.45		
	Scenario D				−1.5	1.45		

CRNA, nurse anesthetist; LS, least square; SE, standard error; *F*, statistic used. ^*∗*^Scenario C and scenario A were attempted twice (before increasing and decreasing heart rate).

**Table 4:** Repeated measures mixed model results for recording setting, music, and training level on oxygen saturation estimation accuracy.

Effect	Scenario	Visual aid	Experience level	Music experience	Adjusted LS mean	SE	*F*	*p* value
Setting	Scenario A				−2.3	0.49	8.79	<0.0001
	Scenario B				−2.6	0.49		
	Scenario C				−3.3	0.49		
	Scenario D				−0.8	0.49		

Visual aid		Yes			−2.3	0.42	0.20	0.6542
		No			−2.2	0.42		

Experience level			Attending		−2.4	0.77	2.94	0.0410
			Resident		−1.0	0.50		
			CRNA		−1.7	0.55		
			Intern		−3.9	0.92		

Music experience				Yes	−2.3	0.62	0.01	0.9207
				No	−2.23	0.38		

Visual aid × setting^*∗*^	Scenario C	Yes			−2.6	0.60	0.49	0.6869
	Scenario B				−2.6	0.60			
	Scenario C				−3.6	0.60			
	Scenario D				−0.6	0.60			
	Scenario A	No			−2.1	0.60			
	Scenario B				−2.6	0.60			
	Scenario A				−3.0	0.60			
	Scenario D				−1.1	0.60			

CRNA, nurse anesthetist; LS, least square; SE, standard error; *F*, statistic used. ^*∗*^Scenario C and scenario A were attempted twice (before increasing heart rate and decreasing heart rate and oxygen saturation).

## Data Availability

The data used to support the findings of this study are available from the corresponding author upon request.

## Additional Points

- Estimation of vital signs by auditory cues is helpful when the anesthesia provider is performing procedures in the operating room.
- Anesthesia providers' ability to estimate heart rate and oxygen saturation by auditory cues is unsatisfactory.
- Novice anesthesia providers exhibit the worse ability to estimate heart rate and oxygen saturation by pitch sound.

## Supplementary Materials

Supplementary Materials Video displays the instructions for the participants to identify baseline and changes in auditory cues for heart rate and pulse oximetry. During the video, the tones change as the two variables of study change.
